# The Impact of Migraine Disease on Work Productivity and Quality of Life Among the Adults in Riyadh, Saudi Arabia

**DOI:** 10.7759/cureus.27733

**Published:** 2022-08-06

**Authors:** Rahaf F Alkahtani, Shawg S Alrumaih, Sarah S Algezlan, Rahaf R Almutairi, Basma A Alturki, Raghad M Alanazi, Fahad A Alateeq

**Affiliations:** 1 Medicine, Imam Muhammad ibn Saud Islamic University, Riyadh, SAU; 2 Medicine, Imam Muhammad Ibn Saud Islamic University, Riyadh, SAU; 3 Medicine, Al Imam Muhammad Ibn Saud Islamic University, Riyadh, SAU; 4 Medical Education and Family Medicine, Al Imam Muhammad Ibn Saud Islamic University, Riyadh, SAU

**Keywords:** quality of life, id migraine™, neurological diseases, work productivity, migraine

## Abstract

Background: Migraine is a chronic unilateral headache associated with nausea and vomiting. According to the World Health Organization, it is listed as the 19th disabling disease. Multiple studies found an inverse relationship between the frequency of the attacks and the low quality of life score. Roughly, one-third of migraine attacks occur during workdays, with a higher incidence of reduced productivity and missed days among chronic patients (>15 headache days per month).

Objective: The objective of this article was to determine the migraine impact on work productivity and quality of life in Riyadh's general population in Saudi Arabia.

Materials and methods: A cross-sectional study was conducted among participants suspected or diagnosed with migraine in Riyadh, Saudi Arabia. The survey measured the quality of life with the effect of migraine headaches by the Migraine Specific Quality of Life Questionnaire (MSQ). Patients suspected of migraines were asked to complete the ID Migraine™ three-item screening test.

Results: In this study, we were able to collect data from 223 patients diagnosed with migraine. Among the participants, 99.1% of them were Saudi Arabian, and 93.7% were females. Moreover, 33.6% of the participants were included in the study upon their self-report and 66.4% according to the ID migraine assessment. The mean scores of limitations of the patients’ performance, interrupting normal activities, and emotions were 51.83, 57.11, and 59.94, respectively.

Conclusion: Our study confirmed the results reported in previous studies that migraine has a negative impact on the quality of life of the patients and their ability to work. An awareness program should be conducted to increase the awareness of the importance of the early diagnosis of migraine.

## Introduction

Migraine is a chronic episodic neurological disease [1]. It presents as unilateral, pulsatile pain associated with nausea and vomiting. The attack’s duration ranges from a minimum of four hours up to a maximum of 72 hours [2,3]. It usually affects the adult age group between 25-55 years [4,5]. Migraine is three times more prevalent among women (18%) when compared to men (6%), which might be related to hormonal imbalance [6,7]. It is estimated to affect 12% of the population worldwide and 26% in Saudi Arabia [8,9]. The majority of migraine attacks are associated with functional impairments [10-12], which induce a negative physical and psychological effect [13]. In fact, Buse et al. found that more than half of the people suffering from migraine need bed rest [11]. Those attacks/consequences ranked migraine as the 19th most disabling disease worldwide according to the World Health Organization [14] resulting in a significantly lower quality of life (QOL) than patients with chronic conditions such as diabetes mellitus, arthritis, and hypertension [15-20]. The QOL was defined by the World Health Organization as “an individual’s perception of their position in life in the context of the culture and value systems in which they live and in relation to their goals, expectations, standards, and concerns” [21]. Multiple studies found an inverse relationship between the frequency of the attacks and the low QOL score [19,22,23].

Previous studies showed a positive impact on the QOL in patients with effective migraine therapy [24-27]. However, approximately half of the migraineurs were found to be undiagnosed [24,28], which may contribute to the loss in workers' productivity since no treatment is used. Measuring the QOL and disability is important for determining the burden and management of migraine [9,29]; most of the studies reported the disability by calculating the time lost due to migraine at the workplace, school, or even in doing daily activities [30-34]. Roughly one-third of migraine attacks occur during workdays [35], with a higher incidence of reduced productivity and missed days among chronic (>15 headache days per month) and severe migraine patients (grades III and IV) [29,36]. The estimated average number of missing work due to migraine is four days per year [1], while there is a loss of productivity of 6.7 hours/week per employee [22]. The relation between migraine and decreased work productivity is well-supported in the literature in western countries; however, it is yet to be investigated in our community. Therefore, we aim to investigate the relationship between migraine and work productivity in the population of Riyadh, Saudi Arabia. Assessing the effect of migraine on productivity and determining the factors and triggers of the attacks among the population could aid in increasing the QOL and work productivity by increasing awareness about the treatment options, leading to an improvement in managing the condition.

## Materials and methods

This is a cross-sectional study that was conducted among participants who are suspected or diagnosed with migraine by a self-administrated online survey that was published through social media platforms. Participating in the study was completely voluntary, noticing that all participants were informed with consent to join the study before the completion of the questionnaire. The inclusion criteria included patients aged 18 years and above diagnosed with migraine for more than one year or suspected to have migraine and confirmed by the ID Migraine™ questionnaire. Patients with any neurological or cardiovascular disorders and pregnant women were excluded from the study. The sample size was found to be 288 for this cross-sectional study, which is based on a response rate of 50%, a confidence interval of 95%, and a margin of error of 5%. The following formula was used to calculate the sample size:

Sample size = $Z \frac{1-\alpha}{2} 2\frac{SD^{2}}{D^{2}}$, where $\frac{Z1-\alpha }{2}$ is the standard normal variate (5% type 1 error), SD is the standard deviation of the variable, and d is the absolute error.

An adjustment to 300 responses was taken as the sample size to compensate for any missing data or mismatching of the inclusion criteria. This study was approved by the institutional review board (IRB) Ethics Committee of Al Imam Muhammad Ibn Saud University in Riyadh with the approval number of 233/2022.

The survey was divided into the following sections: (1) sociodemographic, including the gender, age, marital status, smoking status, comorbidities, and occupation. Furthermore, an Arabic version of the Migraine Specific Quality of Life Questionnaire (MSQ) was used to measure the QOL with the effect of migraine headaches. It consists of 14 items; the first seven items assess the limitations of migraine on patients’ performance; the next four items assess how it is interrupting normal activities, and the last three items assess the impact of migraine on emotions [37]. Patients that were suspected to have migraine will be asked to complete the ID Migraine™, and a three-item screening test helps to confirm the diagnosis of migraine [38]. 

Microsoft Excel (Microsoft Corporation, Redmond, Washington) was used for data entry, while SPSS software version 26 (IBM Corp., Armonk, NY) was used for data analysis. Frequency and percent were used for the description of categorical variables, while mean and standard deviation were used for the description of the ongoing variables including the subscales of the MSQ tool. To assess the scores of the subscales of MSQ, each response was coded as follows: always as (1) point, almost as (2), pretty as (3), sometimes as (4), rarely (5), and never as (6). For each subscale, a formula was provided by the designers of the questionnaire as follows: For the first subscale of limitation of patients’ performance, the equation is $(Score-7)\times \frac{100}{35}$; for interrupting normal activity subscales, the equation is $(Score-4)\times \frac{100}{20}$, and for the effect on emotions subscale, the equation is $(Score-3)\times \frac{100}{15}$.

T-tests and chi-tests were used to assess the relationship between the demographic factors and MSQ subscales. All statements were considered significant if the p-value is lower or equal to 0.05.

## Results

In this study, we were able to collect data from 386 participants for our questionnaire; however, 163 participants were excluded for the next reasons; eight participants refused to participate, three participants were younger than 18 years, 10 patients were pregnant, 22 participants have neurological or cardiovascular conditions, and 102 participants were not diagnosed with migraine using our tool. Among the participants, 99.1% of them were Saudi Arabians, and 93.7% were females. We found that 36.8% of the participants were between 18 and 29 years old, while 30.5% were between 40 and 49 years old, and 17.5% were between 30 and 39 years old. Moreover, we found that 56.5% of the participants were married, while 35.9% were single, and 3.6% of them were smokers. Furthermore, 32.3% of the participants reported that they are governmental employees, while 29.6% were students, and 23.8% were unemployed. Considering the participants having any other chronic disorders, 59.2% indicated having no other chronic disorders, while obesity is reported in 7.2% of them, followed by stress and depression (6.7%) and diabetic mellitus (5.4%). Moreover, 49.3% of the participants reported having a monthly income of lower than 5000 SR (Saudi Riyal); 57.8% reported having university-level education, and 27.8% have secondary school education (Table 1).

**Table 1:** The demographic factors of the patients (n = 223) IBS: Irritable bowel syndrome.

		N = 223	%
Nationality	Saudi	221	99.1%
	Non-Saudi	2	0.9%
Gender	Male	14	6.3%
	Female	209	93.7%
Age (years)	18-29	82	36.8%
	30-39	39	17.5%
	40-49	68	30.5%
	50-59	29	13.0%
	60 and above	5	2.2%
Marital status	Single	80	35.9%
	Married	126	56.5%
	Widow	5	2.2%
	Divorced	12	5.4%
Smoking	Yes	8	3.6%
	No	215	96.4%
Profession	Student	66	29.6%
	Governmental employee	72	32.3%
	Private employee	14	6.3%
	In healthcare	4	1.8%
	Unemployed	53	23.8%
	Retired	14	6.3%
Having chronic disorder	No	132	59.2%
	IBS	18	8.1%
	Diabetic mellitus	12	5.4%
	Stress and depression	15	6.7%
	Obesity	16	7.2%
	Thyroid disorders	0	0.0%
	Other	30	13.5%
Monthly income	<5000 SR	110	49.3%
	6000-10,000 SR	53	23.8%
	>10000 SR	60	26.9%
Educational level	Intermediate	6	2.7%
	Secondary	62	27.8%
	Diploma	17	7.6%
	University	129	57.8%
	Higher education	9	4.0%

**Figure 1:**
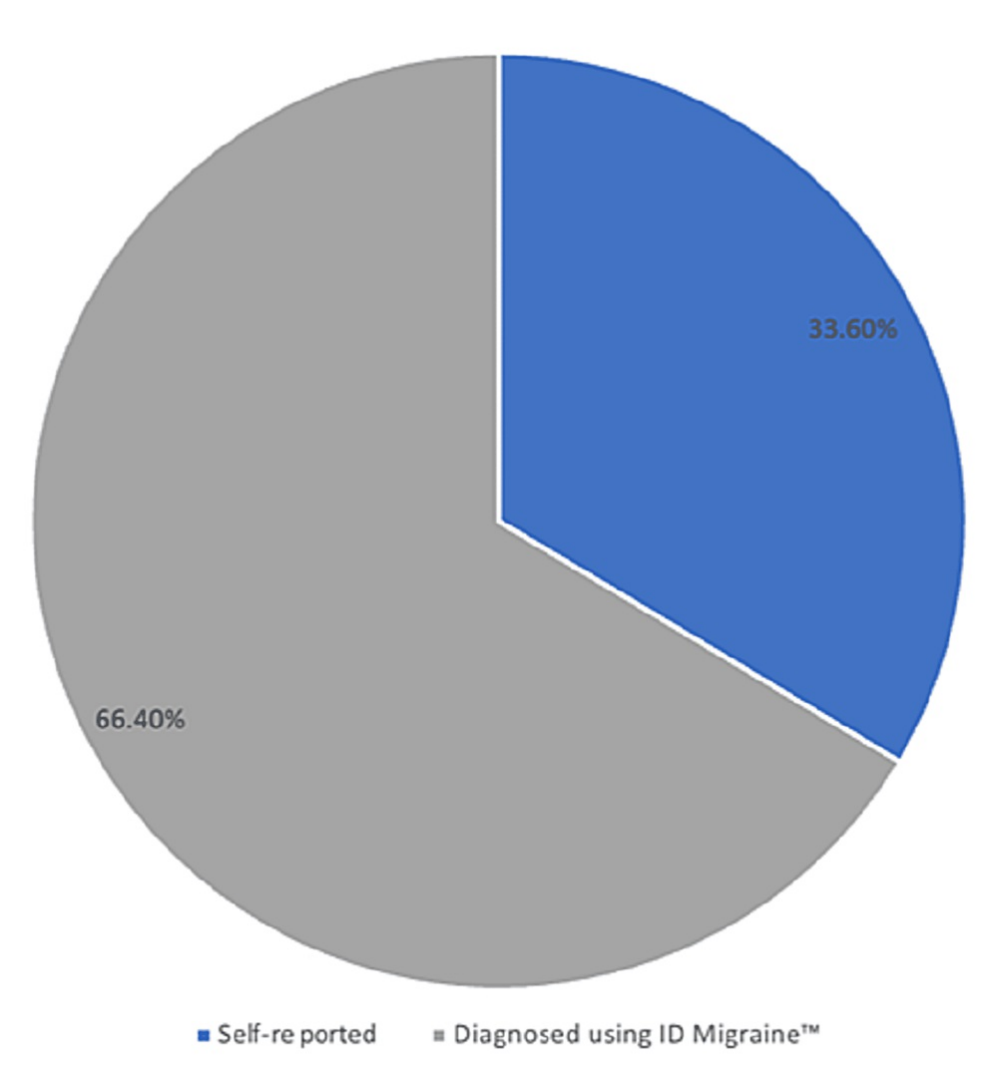
The distribution of the patients according to the diagnosis criteria

In this study, we used two tools for the diagnosis of migraine among the participants; self-reported diagnosis and use of ID Migraine™, where 33.6% of the participants were included in the study upon their self-report and 66.4% according to the ID migraine assessment (Figure 1).

Moreover, 13.9% of the participants were diagnosed with migraine more than five years ago, and 9% were diagnosed three to five years ago. The MSQ assesses the QOL across three subscales: limitations of patients’ performance, interrupting normal activities, and emotions with a scale between 0% and 100% where a higher score indicated better QOL. The mean of the three subscales among the total sample were 51.83, 57.11, and 59.94, respectively. According to Table 2, we found that gender had no significant impact on the QOL because of migraine; however, females reported slightly better QOL on all scales (p = 0.836, 0.621, and 0.651). Moreover, we found that younger participants had the least QOL considering the effect of migraine on their emotions (p = 0.001). Also, the most common effect of migraine on emotions was found among widow patients (p = 0.001). Smoking was the only factor that affects the three subscales of the QOL of patients with migraine where smokers reported lower QOL in all aspects than non-smokers. Moreover, the profession of the patients has a significant effect on the impact of migraine on the emotions subscale where retired patients showed the highest QOL. The education level of the patients has no effect on the QOL of the patients with migraine (Table 2).

**Table 2:** The quality of life among the patients with migraine using Migraine Specific Quality of Life Questionnaire and their demographic factors * p-value ≤ 0.05.

		Limitations on patients’ performance	Interrupting normal activities	Emotions
Mean of the total sample		51.83	57.11	59.94
Gender	Male	50.41	53.57	56.67
	Female	51.93	57.34	60.16
	P-value	0.836	0.621	0.651
Age (years)	18-29	51.11	59.57	51.06
	30-39	47.03	53.21	59.66
	40-49	51.47	54.85	64.22
	50-59	59.51	58.28	73.56
	60 and above	61.71	71.00	70.67
	P-value	0.349	0.525	0.001*
Marital status	Single	49.71	58.06	49.83
	Married	53.38	56.55	65.08
	Widow	53.71	66.00	77.33
	Divorced	49.05	52.92	66.11
	P-value	0.781	0.816	0.000*
Smoking	Yes	33.21	34.38	40.00
	No	52.53	57.95	60.68
	P-value	0.043*	0.017*	0.039*
Profession	Student	48.10	58.41	48.28
	Governmental employee	51.98	55.42	62.41
	Private employee	47.76	53.21	68.10
	In healthcare	61.43	62.50	61.67
	Unemployed	54.99	58.21	65.16
	Retired	58.16	57.86	73.81
	P-value	0.596	0.968	0.002*
Monthly income	<5000 SR	50.26	57.50	53.82
	6000-10,000 SR	51.91	53.87	65.41
	>10000 SR	54.67	59.25	66.33
	P-value	0.588	0.574	0.005*
Educational level	Intermediate	52.38	46.67	67.78
	Secondary	50.88	58.06	51.51
	Diploma	54.29	54.71	59.22
	University	51.89	57.95	63.88
	Higher education	52.70	50.00	57.78
	P-value	0.994	0.778	0.065

## Discussion

Many previous studies have shown that migraine patients experience a lower QOL than the general population, and the attack frequency is inversely related to QOL score [17,19]. Moreover, some studies also showed that the effective treatment of migraine has a positive effect on health-related QOL [24-27]. The aim of this study was to investigate the relationship between migraine and work productivity in the population of Riyadh, Saudi Arabia.

In this study, we included 223 patients with migraine where 33.6% were from patients’ reports and 66.4% according to the ID migraine assessment. The ID migraine assessment tool consisted of three questions where only participants who answered "Yes" for two or three questions were diagnosed with migraine and included in the study, while those who answered "Yes" for one question or "No" for the three questions were excluded from the study. This indicates that 66.4% of patients with migraine did not know that they had migraine or did not tend to physicians in order to have a correct diagnosis. The ID migraine assessment is a validated questionnaire for the diagnosis of migraine [38]. In a previous study conducted among employees in two different banks, the authors found that 47.2% of them were diagnosed with migraine using the ID migraine assessment tool [39]. Our results are similar to the results reported in European countries where migraine is one of the conditions which remains underdiagnosed [40]. These findings ensure the need to increase the awareness of public populations with recurrent headaches to seek appropriate diagnosis and, hence, care [40].

In this study, we found that patients with migraine were mostly females and those aged between 30 and 49 years. Similar results had been reported in a previous study which showed that the prevalence of migraine peaks at their prime working age (31-40 years) [41]. The reason for the high prevalence of migraine during this period is that career and family responsibilities started to appear. This is typically most crucial and implies that migraines not only pose immense economic burdens on the patients but also had a negative impact on the harmony of the patients’ social and family life. Moreover, similar to this study, a higher prevalence of migraine among females compared to males is also reported in some other studies [41,42]. The reason for the high prevalence of migraine among females compared to males is the difference in the physiological and hormonal influences [42]. This result suggests the need for a gender-based strategy to promote the prevention and care of migraine risks.

The present study showed that migraine has a substantial negative impact on participants’ QOL and ability to work. The mean of the three subscales, limitations of patients’ performance, interrupting normal activities, and emotions, among the total sample, was 51.83, 57.11, and 59.94, respectively. This is consistent with other research [34]. This clearly demonstrates that migraine caused impairments in work productivity and resulted in a substantial occupational disability. In a previous study conducted among healthcare workers in primary healthcare centers in Abha, Saudi Arabia, via self-administered questionnaires such as Migraine Disability Assessment (MIDAS) and World Health Organization Quality of Life (WHOQOL-BREF), the authors found that MIDAS Mean ± SD was 12 ± 10.6, and 54.9 ± 7.4 for health-related quality of life (HRQOL), and the authors also found that smoking was significantly associated with HRQOL (p = 0.037), which is similar to our results where smoking is the only factor that affects the QOL among patients [43]. Smoking has a negative impact on the QOL even among normal populations. Non-smokers had an average probability of having a higher QOL than smokers [44]. Moreover, lower QOL and higher prevalence of depression were associated with higher odds of smoking initiation and lower odds of successful smoking cessation [45]. A study conducted by Alharbi and AlAteeq showed that the mean scores of the QOL restrictive, preventive, and emotional domains were 51.8 ± 19, 54 ± 18, and 46.3 ± 23.4, respectively, which is quite similar to our results [9]. However, the same study showed that decreased QOL scores were especially seen among young patients, patients with repeated migraine attacks, patients who do not use prophylactic treatment, and individuals diagnosed with a chronic disease [9].

This study had some limitations including dependence on a self-report questionnaire for the diagnosis of migraine, which may lead to some personal bias. Moreover, depending on the self-reported tool for the diagnosis of migraine could not be used without clinical examinations and diagnosis. Therefore, we suggest that further investigations depending on clinical examination are required. 

## Conclusions

In conclusion, our study confirmed the results reported in previous studies that migraine has a negative impact on the QOL of the patients and their ability to work. Therefore, awareness programs should be conducted to increase the awareness of the importance of the early diagnosis of migraine.

## Appendices

**Table 3:** Questionnaire

Demographic factors
1) Age (years)
<18
18-29
30-39
40-49
50-59
60+
2) Nationality
Saudi
Non-Saudi
3) Gender
Female
Male
4) What is your marital status?
Single
Married
Divorced
Widowed
5) If married, are you pregnant?
Yes
No
6) Do you smoke?
Yes
No
7) What is your occupation?
Student
Healthcare sector
Student
Healthcare sector
Government sector
Freelance
Retired
Unemployed
Others
8) Do you have any chronic diseases?
Cardiovascular diseases
Neurological diseases
Hypertension
Diabetes
Obesity
Thyroid disorders
Depression
Anxiety
Irritable bowel syndrome
Others
None
9) Which of these describe your personal income?
5000 SR or less
6000–10000 SR
11000 and more SR
10) What is the highest degree or level of education you have completed?
No education
Elementary
Intermediate
High school
Diploma
University
Master’s degree or higher
11) Are you diagnosed with migraine? Yes or no
12) If yes, for how long are you diagnosed with migraine?
Less than 12 months
1-2 years
3-5 years
More than 5 years
If not diagnosed with migraine, please answer the following screening test: During the last three months, did you have the following with your headache?
13) You felt nauseated or sick to your stomach?
Yes
No
14) Light bothered you (a lot more when you didn’t have headaches)?
Yes
No
15) Your headaches limited your ability to work study or do what you need to do for at least one day?
Yes
No
MSQ: Migraine Specific Quality of Life Questionnaire
While answering the following questions, please think about all migraine attacks you may have had in the past four weeks.
16) How often have migraines interfered with how well you dealt with family, friends, and others who are close to you?
Always
Almost
Pretty
Sometimes
Rarely
Never
17) How often have migraines interrupted with your leisure time activities such as reading or exercising?
Always
Almost
Pretty
Sometimes
Rarely
Never
18) How often have you had difﬁculty in performing work or other daily activities?
Always
Almost
Pretty
Sometimes
Rarely
Never
19) How often have migraines kept you from getting as much accomplished as you normally do at work or at home?
Always
Almost
Pretty
Sometimes
Rarely
Never
20) How often have migraines limited your ability to work or do other activities as carefully as you usually do them?
Always
Almost
Pretty
Sometimes
Rarely
Never
21) How often have you had to cancel or delay work or social activities because you were exhausted?
Always
Almost
Pretty
Sometimes
Rarely
Never
22) How often have migraines left you with limited energy levels?
Always
Almost
Pretty
Sometimes
Rarely
Never
23) How often have you had to stop work or other activities?
Always
Almost
Pretty
Sometimes
Rarely
Never
24) How often have you needed the help of other people in handling routine tasks such as everyday household chores, doing necessary business, shopping, or caring for others when you had a migraine attack?
Always
Almost
Pretty
Sometimes
Rarely
Never
25) How often have you avoided social or family activities to treat your migraine attacks?
Always
Almost
Pretty
Sometimes
Rarely
Never
26) How often has it been difﬁcult for you to go to social events such as parties?
Always
Almost
Pretty
Sometimes
Rarely
Never
27) How often have you felt fed up or frustrated?
Always
Almost
Pretty
Sometimes
Rarely
Never
28) How often have you felt like you were a burden on others?
Always
Almost
Pretty
Sometimes
Rarely
Never
29) How often have you been afraid of letting others down?
Always
Almost
Pretty
Sometimes
Rarely
Never

## References

[1] Burton WN, Landy SH, Downs KE, Runken MC (2009). The impact of migraine and the effect of migraine treatment on workplace productivity in the United States and suggestions for future research. Mayo Clin Proc.

[2] Bigal ME, Liberman JN, Lipton RB (2006). Age-dependent prevalence and clinical features of migraine. Neurology.

[3] Yazdanparast M, Abrishamizadeh AA, Mahboobi H (2013). Prevalence of and factors associated with migraine in medical students at BandarAbbas, Southern Iran, in 2012. Electron Physician.

[4] Stang PE, Osterhaus JT (1993). Impact of migraine in the United States: data from the National Health Interview Survey. Headache.

[5] Lipton RB, Stewart WF, von Korff M (1997). Burden of migraine: societal costs and therapeutic opportunities. Neurology.

[6] Lipton RB, Scher AI, Kolodner K, Liberman J, Steiner TJ, Stewart WF (2002). Migraine in the United States: epidemiology and patterns of health care use. Neurology.

[7] Lucchetti G, Peres MF (2011). The prevalence of migraine and probable migraine in a Brazilian favela: results of a community survey. Headache.

[8] Lipton RB, Bigal ME, Diamond M, Freitag F, Reed ML, Stewart WF (2007). Migraine prevalence, disease burden, and the need for preventive therapy. Neurology.

[9] AlHarbi FG, AlAteeq MA (2020). Quality of life of migraine patients followed in neurology clinics in Riyadh, Saudi Arabia. J Family Community Med.

[10] Leonardi M, Steiner TJ, Scher AT, Lipton RB (2005). The global burden of migraine: measuring disability in headache disorders with WHO's Classification of Functioning, Disability and Health (ICF). J Headache Pain.

[11] Buse DC, Rupnow MF, Lipton RB (2009). Assessing and managing all aspects of migraine: migraine attacks, migraine-related functional impairment, common comorbidities, and quality of life. Mayo Clin Proc.

[12] Brandes JL (2009). Migraine and functional impairment. CNS Drugs.

[13] Diamond M (2007). The impact of migraine on the health and well-being of women. J Womens Health (Larchmt).

[14] Koehler PJ, van de Wiel TW (2001). Aretaeus on migraine and headache. J Hist Neurosci.

[15] Tulen JHM, Stronks DL, Bussmann JBJ, Pepplinkhuizen L, Passchier J (2000). Towards an objective quantitative assessment of daily functioning in migraine: a feasibility study. Pain.

[16] Bigal ME, Fernandes LC, Moraes FA, Bordini CA, Speciali JG (2000). [Migraine prevalence and impact in employees of the clinical hospital of the medical school of Ribeirão Preto-USP]. Arq Neuropsiquiatr.

[17] Lipton RB, Liberman JN, Kolodner KB, Bigal ME, Dowson A, Stewart WF (2003). Migraine headache disability and health-related quality-of-life: a population-based case-control study from England. Cephalalgia.

[18] Magnusson JE, Becker WJ (2003). Migraine frequency and intensity: relationship with disability and psychological factors. Headache.

[19] Terwindt GM, Ferrari MD, Tijhuis M, Groenen SM, Picavet HS, Launer LJ (2000). The impact of migraine on quality of life in the general population: the GEM study. Neurology.

[20] Osterhaus JT, Townsend RJ, Gandek B, Ware JE Jr (1994). Measuring the functional status and well-being of patients with migraine headache. Headache.

[21] Nakane Y, Tazaki M, Miyaoka E (1999). WHOQOL. Iryo To Shakai.

[22] Selekler HM, Gökmen G, Alvur TM, Steiner TJ (2015). Productivity losses attributable to headache, and their attempted recovery, in a heavy-manufacturing workforce in Turkey: implications for employers and politicians. J Headache Pain.

[23] Martelletti P, Schwedt TJ, Lanteri-Minet M (2018). My migraine voice survey: a global study of disease burden among individuals with migraine for whom preventive treatments have failed. J Headache Pain.

[24] Mushet GR, Miller D, Clements B, Pait G, Gutterman DL (1996). Impact of sumatriptan on workplace productivity, nonwork activities, and health-related quality of life among hospital employees with migraine. Headache.

[25] Cohen JA, Beall D, Beck A (1999). Sumatriptan treatment for migraine in a health maintenance organization: economic, humanistic, and clinical outcomes. Clin Ther.

[26] Jhingran P, Cady RK, Rubino J, Miller D, Grice RB, Gutterman DL (1996). Improvements in health-related quality of life with sumatriptan treatment for migraine. J Fam Pract.

[27] Lofland JH, Johnson NE, Batenhorst AS, Nash DB (1999). Changes in resource use and outcomes for patients with migraine treated with sumatriptan: a managed care perspective. Arch Intern Med.

[28] Lipton RB, Diamond S, Reed M, Diamond ML, Stewart WF (2001). Migraine diagnosis and treatment: results from the American migraine study II. Headache.

[29] Shaik MM, Hassan NB, Tan HL, Gan SH (2015). Quality of life and migraine disability among female migraine patients in a tertiary hospital in Malaysia. Biomed Res Int.

[30] Osterhaus JT, Gutterman DL, Plachetka JR (1992). Healthcare resource and lost labour costs of migraine headache in the US. Pharmacoeconomics.

[31] Stewart WF, Lipton RB, Simon D (1996). Work-related disability: results from the American migraine study. Cephalalgia.

[32] Michel P, Dartigues JF, Lindoulsi A, Henry P (1997). Loss of productivity and quality of life in migraine sufferers among French workers: results from the GAZEL cohort. Headache.

[33] Von Korff M, Stewart WF, Simon DJ, Lipton RB (1998). Migraine and reduced work performance: a population-based diary study. Neurology.

[34] Edmeads J, Findlay H, Tugwell P, Pryse-Phillips W, Nelson RF, Murray TJ (1993). Impact of migraine and tension-type headache on life-style, consulting behaviour, and medication use: a Canadian population survey. Can J Neurol Sci.

[35] Stewart WF, Wood GC, Razzaghi H, Reed ML, Lipton RB (2008). Work impact of migraine headaches. J Occup Environ Med.

[36] Munakata J, Hazard E, Serrano D (2009). Economic burden of transformed migraine: results from the American Migraine Prevalence and Prevention (AMPP) study. Headache.

[37] Chang HY, Jensen MP, Yang CC, Lai YH (2019). Migraine-specific quality of life questionnaire Chinese version 2.1 (MSQv2.1-C): psychometric evaluation in patients with migraine. Health Qual Life Outcomes.

[38] Lipton RB, Dodick D, Sadovsky R, Kolodner K, Endicott J, Hettiarachchi J, Harrison W (2003). A self-administered screener for migraine in primary care: the ID migraine validation study. Neurology.

[39] Wong LP, Alias H, Bhoo-Pathy N, Chung I, Chong YC, Kalra S, Shah ZU (2020). Impact of migraine on workplace productivity and monetary loss: a study of employees in banking sector in Malaysia. J Headache Pain.

[40] Patwardhan M, Coeytaux RR, Deshmukh R, Samsa G (2007). What is the impact of physician communication and patient understanding in the management of headache?. Neuropsychiatr Dis Treat.

[41] Burton WN, Conti DJ, Chen CY, Schultz AB, Edington DW (2002). The economic burden of lost productivity due to migraine headache: a specific worksite analysis. J Occup Environ Med.

[42] Peterlin BL, Gupta S, Ward TN, Macgregor A (2011). Sex matters: evaluating sex and gender in migraine and headache research. Headache.

[43] Alfaifi FJS, Qasim MY, Al-Harban AM, Alqahtani SSA, Alshahrani NMS (2021). Prevalence, determinants and impact of migraine on quality of life of healthcare workers at primary healthcare centers in Abha City, Saudi Arabia. Middle East J Fam Med.

[44] Cheng X, Jin C (2022). The association between smoking and health-related quality of life among Chinese individuals aged 40 years and older: a cross-sectional study. Front Public Health.

[45] Goldenberg M, Danovitch I, IsHak WW (2014). Quality of life and smoking. Am J Addict.

